# Sex differences in addiction: gonadal hormones and substance use effects in women

**DOI:** 10.1192/j.eurpsy.2024.91

**Published:** 2024-08-27

**Authors:** F. Fonseca

**Affiliations:** ^1^Addiction Section, Hospital del Mar; ^2^Addiction Research Group, Hospital del Mar Research Institute; ^3^Medicine and Life Sciences, Universitat Pompeu Fabra, Barcelona, Spain

## Abstract

**Abstract:**

Substance use disorders (SUD) affect differentially women and men. Although the prevalence has been reported higher in men, those women with addictive disorders present a more vulnerable profile and are less likely to enter treatment than men. The aim of this presentation is to present an overview of how gonadal hormones may influence in response to substances, clinical differences in the addictive disorders and implications in treatment response.Ovarian steroid hormones (estrogen, progesterone), the metabolites of progesterone, and negative allosteric modulators of the gamma-aminobutyric acid A (GABA-A) receptor, such as dehydroepiandrostenedione (DHEA) may influence the behavioral effects of drugs.

**Disclosure of Interest:**

None Declared

